# Supplementary material for: Comparative Mapping of the Macrochromosomes of Eight Avian Species Provides Further Insight into Their Phylogenetic Relationships and Avian Karyotype Evolution
Source: Cells. 2021 Feb 9;10(2):362. doi: 10.3390/cells10020362 (PMC7916199; doi:10.3390/cells10020362)
Supplement: Supplementary file 1 [file cells-10-00362-s001.pdf]

# Supplementary Material

## Supplementary Note 1: Correlation analyses

*1.1. Simple correlation*

Let us consider the main characteristics of genome/karyotype differences and chromosomal rearrangements in the eight avian species studied that were summarized in Supplementary Table SN1. We selected the interspecies BAC hybridisation success rate as a major physical indicator of genome 'kinship'/divergence in this dataset taking into account that if the success rate for a particular bird genome is lower, this species is evolutionarily more divergent (less similar) relative to the chicken. To link the observed diploid number of chromosomes, 2n, to the typical avian karyotype (of 80 chromosomes), we also represented it as a conditional share from 80, i.e., as a ratio 2n/80 (see the 4th column in Supplementary Table SN1). Using Microsoft Excel (Microsoft, Redmond, WA, USA), the respective pairwise Pearson's correlation coefficients, *R*, for the success rate as related to 2n, 2n/80, and intra-, inter- and total chromosomal changes ranged between –0.531 and –0.799.

**Supplementary Table SN1.** Characteristics of interspecific FISH hybridization, karyotype, and rearrangements, as well as simple linear correlation between them in the eight studied bird species.

| Species | Success rate | 2n | 2n/80 | Intra rearrangements | Inter rearrangements | Total rearrangements |
|---|---|---|---|---|---|---|
| Chicken | 1.00 | 78 | 0.98 | 3 | 1 | 4 |
| Guinea fowl | 1.00 | 78 | 0.98 | 4 | 2 | 6 |
| Duck | 0.85 | 80 | 1.00 | 8 | 0 | 8 |
| Pigeon | 0.93 | 80 | 1.00 | 11 | 0 | 11 |
| Houbara | 0.88 | 78 | 0.98 | 9 | 0 | 9 |
| Blackbird | 0.78 | 80 | 1.00 | 9 | 1 | 10 |
| Canary | 0.73 | 80 | 1.00 | 8 | 3 | 11 |
| Woodcock | 0.73 | 96 | 1.20 | 8 | 8 | 16 |
| | ***R* (for success rate)** | -0.583 | -0.583 | -0.531 | -0.547 | -0.799 |
| | -0.583 | | ***R* (for 2n/80)** | 0.186 | 0.906 | 0.811 |

For exploring further an association between chromosomal rearrangement patterns, on the one hand, and overall karyotype/genome organisation and divergence time, on the other, for the eight avian species studied, we produced simple correlation graphs using Microsoft Excel (Supplementary Figure SN1a–d). Herewith, instead of particular interspecies FISH success rate (Table 1), percentage values of failed chicken BACs in these avian species were used to reflect relative genome divergence in general and plotted on the *x*-axis. In other words, percentage of failed chicken BACs served as a kind of genome similarity degree calculated in reverse order (by subtracting FISH success rate from 100%) so that the chicken had 0% (instead of 100%) and the other species their respective values that can be considered as a peculiar distance these birds are at from the 'zero' species, i.e., the chicken. As values on the *y*-axis, individual numbers of intra-, inter- or total chromosomal changes or inversions alone (Table 2) were assigned.

As a result, the graphs contained points for the eight bird species, which were approximated by linear correlation functions. Thus, by means of the graphs plotted in this way, we were determining the presence of possible relationships (regularities) between the rearrangement values (Table 2) and the degree of 'kinship' of various bird species relative to the chicken expressed as a percentage of failed chicken BACs. In theory, knowing the degree of 'kinship' with the original species, one may assume what value of total rearrangements a given species may have.

a

b

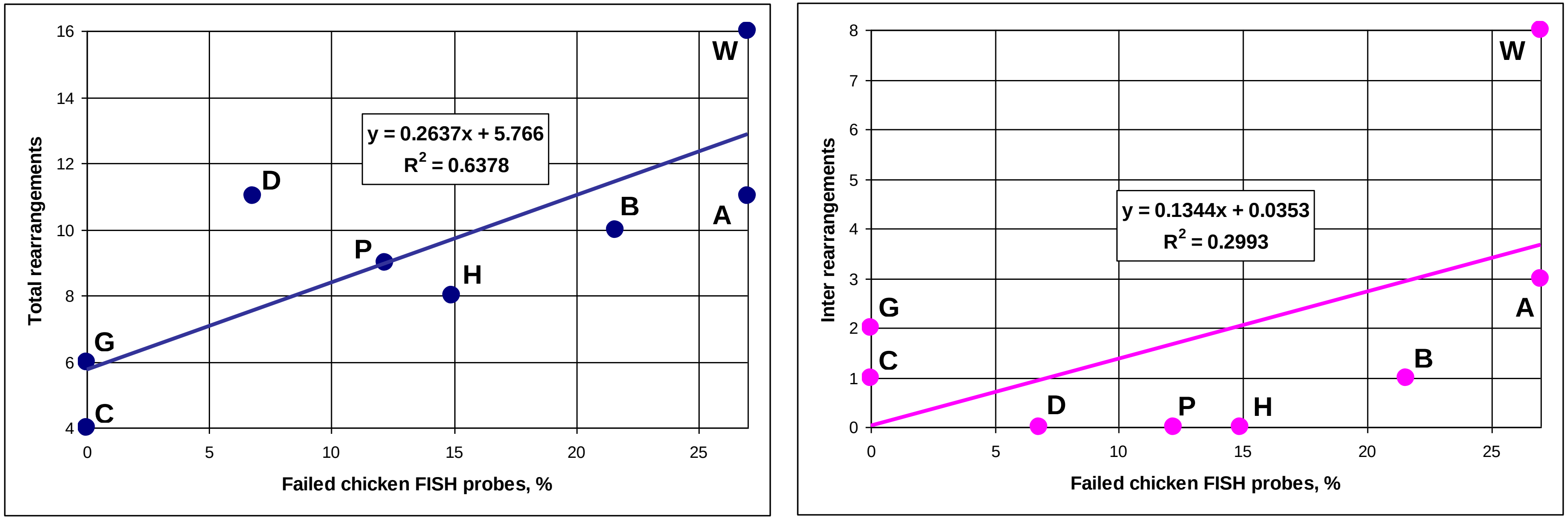

Total rearrangements
Failed chicken FISH probes, %
y = 0.2637x + 5.766
R2 = 0.6378
W
D
B
A
P
H
G
C
Inter rearrangements
Failed chicken FISH probes, %
y = 0.1344x + 0.0353
R2 = 0.2993
W
G
A
C
B
D
P
H

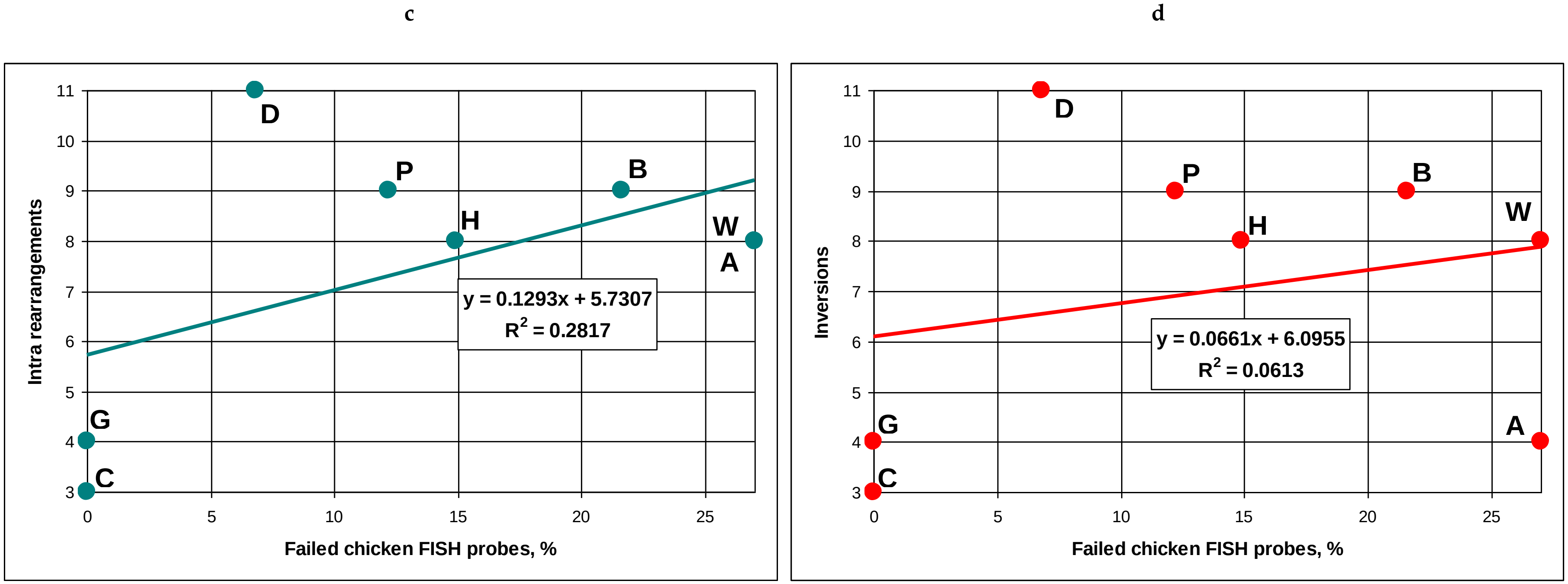


**Supplementary Figure SN1.** Simple correlations between rearrangement metrics including (**a**) total number of rearrangements, (**b**) number of interchromosomal rearrangements, (**c**) number of intrachromosomal rearrangements, and (**d**) inversions, on the one hand, and percentage of failed FISH probes, on the other, in the eight birds studied: C, chicken (*Gallus gallus*); G, helmeted guinea fowl (*Numida meleagris*) ; D, duck (*Anas platyrhynchos*); P, pigeon (*Columba livia*); H, houbara bustard (*Chlamydotis undulata*); B, common blackbird (*Turdus merula*); A, Atlantic canary (*Serinus canaria*); W, Eurasian woodcock (*Scolopax rusticola*).

Along with the correlation analysis for the success rate, similar correlation estimates were obtained for the ratio 2n/80. The respective pairwise coefficients, *R*, for correlation between 2n/80 and other characteristics ranged between 0.186 and 0.906 as can be seen in Supplementary Table SN1. Similarly to the success rate charts (Supplementary Figure SN1a–d), we plotted the graphs describing the dependencies produced for 2n/80 (Supplementary Figure SN2a–d).

Alternatively, we performed a transformative ranking for the following species-specific characteristics: $R_1$, success rate of interspecies FISH hybridization; $R_2$, divergence time (as estimated between the chicken and any other studied bird using TimeTree [39]; Table 1); and $R_3$, ratio of the diploid number of chromosomes of a species (Table 1 and Supplementary Table SN1) to the typical avian karyotype taken as 80 chromosomes, i.e., 2n/80.

As can be seen from Table 1 and Supplementary Table SN1, each of the above three characteristics (factors) has its own specific variability and nature (i.e., magnitude and range) of values. For example, divergence time ($R_2$ factor) for the chicken should be interpreted as 0, while its value for the pigeon, houbara bustard, blackbird, canary and woodcock was equal to 98 million years. If we consider the diploid set of chromosomes (2n) used to calculate the $R_3$ index, we have here, as a rule, only two available variables (78 and 80), and only the woodcock (96) gets out of this row, and so on. In other words, it is rather difficult to integrally generalize such diverse and variable factors into any one indicator. Therefore, as a solution to this problem, it was proposed to transform (normalise) these very different discrete data for the three factors by ranking them. For example, instead of the following available values for 2n/80, which were used as $R_3$:

0.98

0.98

1.00

1.00

0.98

1.00

1.00

1.20

one can assign the following appropriate ranks to each value (in ascending order of the original values from 0 to 8):

1

1

2

2

1

2

2

3

Similar transformations (rankings) were performed for the other two factors. Then, the transformed values for three factors were multiplied for each species, which gave a new integrative indicator. Thus, this integrative genome/divergence index (IGDI) was computed as a product of three single factors using the following formula: IGRI = $R_1 \times R_2 \times R_3$.

Next, a simple linear correlation graph shown in Figure 6a was built using Microsoft Excel for the eight species, where the total number of rearrangements was plotted along the *y*-axis, and IGRI along the *x*-axis. The respective coefficient of determination was $R^2 = 0.8427$, meaning that a simple correlation for this studied dependence ($R$ = 0.9180) was higher than the respective Pearson's correlation coefficients in Supplementary Table SN1.

**a**

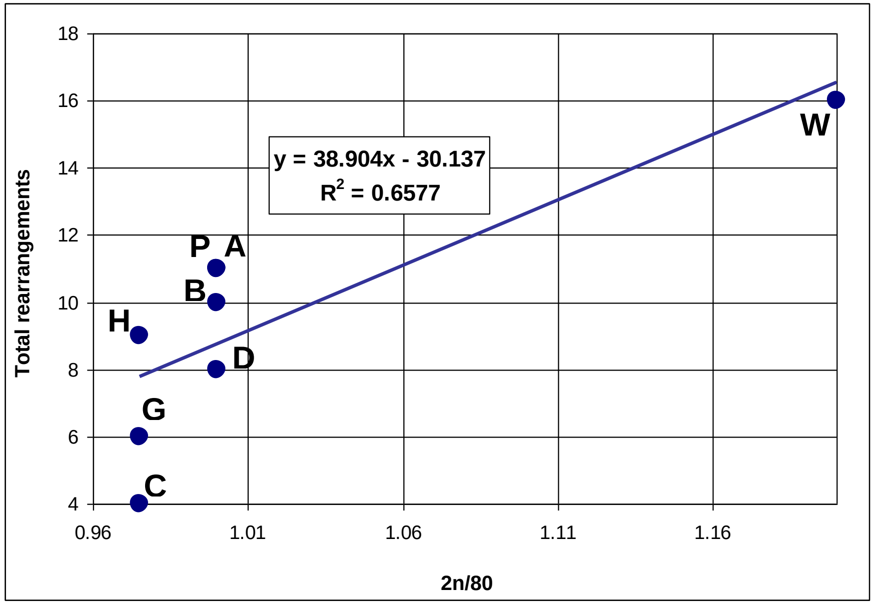
Total rearrangements
2n/80
y = 38.904x - 30.137
R2 = 0.6577
18
16
14
12
10
8
6
4
0.96
1.01
1.06
1.11
1.16
P A B H D G C W

**b**

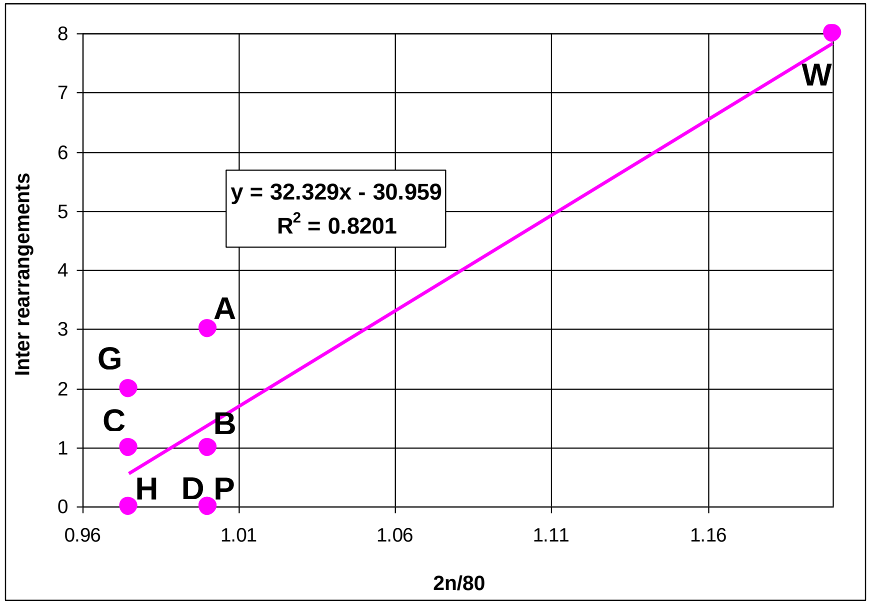
Inter rearragements
2n/80
y = 32.329x - 30.959
R2 = 0.8201
8
7
6
5
4
3
2
1
0
0.96
1.01
1.06
1.11
1.16
A G C B H D P W

c

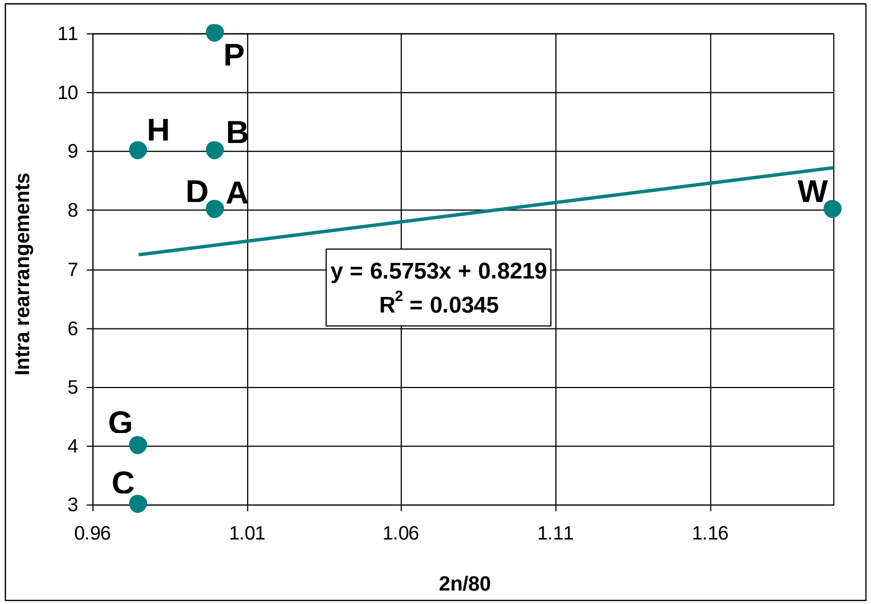


d

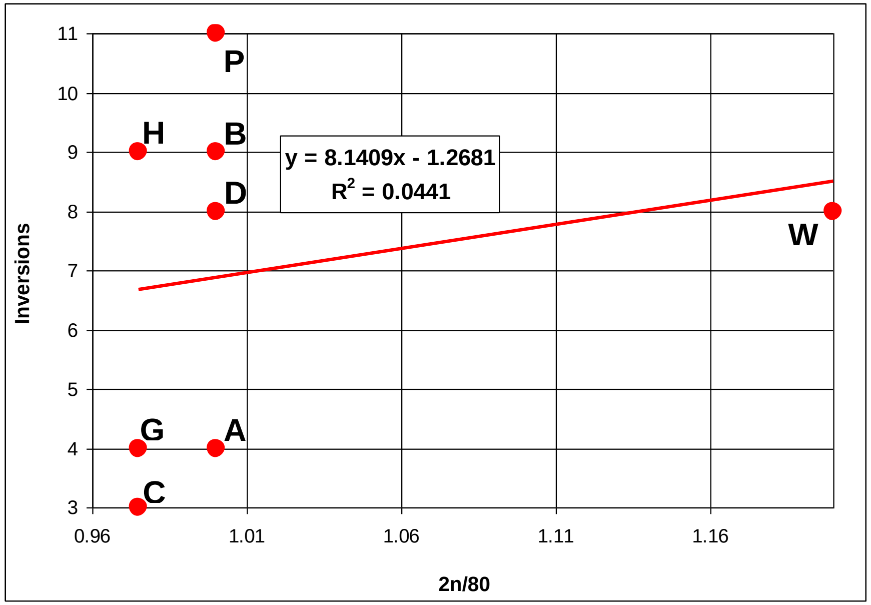


**Supplementary Figure SN2.** Simple correlations between rearrangement metrics including (**a**) total number of rearrangements, (**b**) number of interchromosomal rearrangements, (**c**) number of intrachromosomal rearrangements, and (**d**) inversions, on the one hand, and 2n/80, on the other, in the eight birds studied: C, chicken (*Gallus gallus*); G, helmeted guinea fowl (*Numida meleagris*) ; D, duck (*Anas platyrhynchos*); P, pigeon (*Columba livia*); H, houbara bustard (*Chlamydotis undulata*); B, common blackbird (*Turdus merula*); A, Atlantic canary (*Serinus canaria*); W, Eurasian woodcock (*Scolopax rusticola*).

*1.2. Multiple correlation*

We also tested a different approach by searching for multiple correlation dependence of the total number of rearrangements on the hybridization success rate and 2n/80. Graphically, this can be visualised as a 3D diagram plotted using STATISTICA 5.5 (StatSoft, Inc./TIBCO, Palo Alto, CA, USA) and shown in Figure 6b, that had the following axes:

$x = s$ (success rate changing from 1.00 to 0.73), or VAR2;

$y = k$ (2n/80 ranging between 0.98 and 1.20), or VAR3;

z = $TR$ (total rearrangements), or VAR1.

Approximation of values of the success rate (see Supplementary Table SN1, 2nd column) and 2n/80 (see Supplementary Table SN1, 4th column) yielded two types of formulae:

$$TR = 6.895s^{-1.554}\,k^{1.972}, \quad (1)$$

$R = 0.887$ ($p < 0.01$);

and

$$TR = 5335.457 - 3736.18s - 7448.127k + 3327.59sk + 244.407s^2 + 2292.01k^2, \quad (2)$$

$R = 0.983$ ($p < 0.001$),

where $R$ is the coefficient of correlation between actual data and those obtained as a result of calculation by formula (1) or (2).

Formula (2) is more accurate and can be taken as a basis for describing values of total rearrangements depending on the success rate and 2n/80.

**References**

39 Kumar, S., Stecher, G., Suleski, M., Hedges, S.B. TimeTree: a resource for timelines, timetrees, and divergence times. *Mol. Biol. Evol.* **2017**, *34*, 1812–1819. https://doi.org10.1093/molbev/msx116.

# Supplementary Material

## Supplementary Note 2: PCA, FAC and ALC clustering analyses

To examine relationships between the eight studied avian species as influenced by their genome/karyotype and rearrangement features, the respective charts were plotted using the methods of principal component analysis (PCA), fuzzy analysis clustering (FAC), and average linkage clustering (ALC) based on Euclidean distances. Input dataset for these analyses included four or five characteristics (factors) for interspecific FISH hybridization, karyotype and rearrangements as follows: success rate, diploid number of chromosomes (2n), and numbers of intra-, interchromosomal and total rearrangements (see their appropriate values for each species in Supplementary Table SN1). For data analysing and plotting, R language and libraries for R environment were used. Prior to running the PCA and FAC analyses, the data were transformed (normalized) by scaling [69]. Further, depending on datasets, the appropriate metric was chosen, which was mostly the Euclidean distance, and the clustering method was the average linkage method.

*2.1. PCA analysis*

As a result of performing PCA on the base of four karyotype/rearrangement characteristics and producing the respective score plot, it was found that the success rate and number of total rearrangements contributed to PC1, while the numbers of intra- and interchromosomal rearrangements to PC2 (Figure 7). The change in the success rate factor was inversely proportional to the change in the total rearrangements factor (as can also be suggested from the Supplementary Table SN1 data).

The used four factors had approximately equal degree of influence on differentiation of the eight compared birds. In particular, there were the following two distinct groups (Figure 7): chicken–guinea fowl (Galliformes) and duck–houbara–pigeon–canary–blackbird, the latter being divided into two subgroups, duck–houbara–pigeon (mixed) and canary–blackbird (Passeriformes), while woodcock remarkably differed from the others due to the least hybridization success rate and a large number of inter- and intrachromosomal rearrangements (eight of each type). As can be seen from position of the eight birds on the PCA score plot (Figure 7), the pair chicken–guinea fowl had the greatest possible success rate, and the other birds had respectively lower values. On the other hand, duck, houbara, pigeon, canary, and blackbird had the greatest number of intrachromosomal rearrangements as reflected by their position on the score plot.

If the fifth characteristics, i.e., 2n, was included in the PCA analysis, there was some more pronounced correlation between this factor and intrachromosomal rearrangements, as shown on the PCA score plot in Supplementary Figure S2.1. Overall, this resulted however in somewhat distorted arrangement of the eight species relative to PC1 and PC2, although it retained main features of relationships within this set of birds as observed for the four characteristics (Figure 7).

*2.2. FAC analysis*

Using the same dataset, we applied the FAC method by employing the function `fanny()` from the package `cluster` [70–72] and Dunn's partition coefficient ($F_k$) according to the following formulae:

$$F_k = \sum_{i=1}^{n} \sum_{r=1}^{k} {\mu^2}_{ir} / k$$

where $\sum_{r=1}^{k} {\mu^2}_{ir} / k$ is Dunn coefficient for each observation in a matrix of objects, $\mu$ is membership coefficient, $\mu_{ir}$ is ratio of the membership coefficient of observation $i$ to cluster $r$, $k$ is the number of clusters, $r$ is a selected cluster, and $i$ is an object of observation.

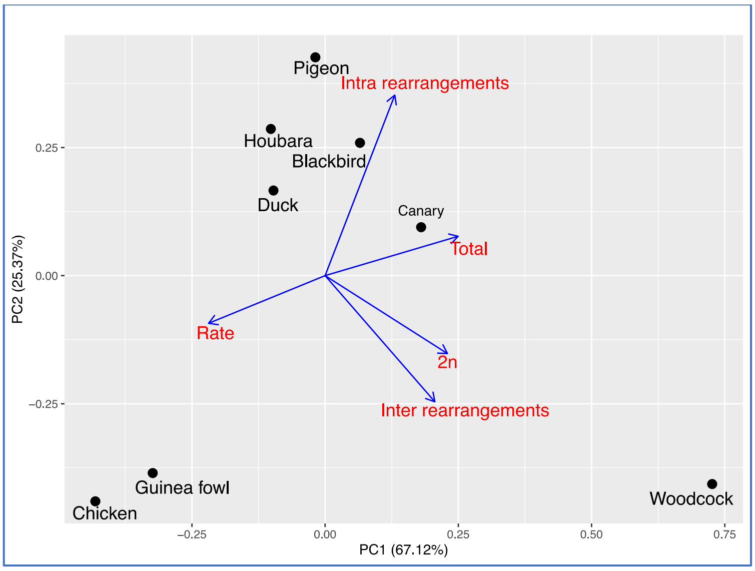


**Supplementary Figure S2.1.** PCA score plot generated for the eight bird species studied using five characteristics: BAC hybridization success rate (Rate), diploid number of chromosomes (2n), and numbers of total (Total), intra- (Intra rearrangements) and interchromosomal (Inter rearrangements) rearrangements.

The above procedure enabled to obtain the respective chart shown in Supplementary Figure S2.2. For the paired objects, i.e., three clusters (1) chicken–guinea fowl, (2) houbara–pigeon, and (3) canary–blackbird, their members had greater Dunn's partition coefficient values, i.e., $0.26 < F_k < 0.87$, as shown on the left diagram in Supplementary Figure S2.2. The appropriate clusters (on the right score plot in Supplementary Figure S2.2) were clearly defined, and those were consistent with the known phylogeny (Figure 1). As far as duck and woodcock are concerned, they had lower Dunn's partition coefficient values, at $F_k < 0.17$, meaning that they were to a smaller degree similar to the three observed distinct clusters. At the same time, they did not have any obvious pair on the score plot, resembling, to some extent and at least for woodcock, their positions in the phylogenetic tree in Figure 1.

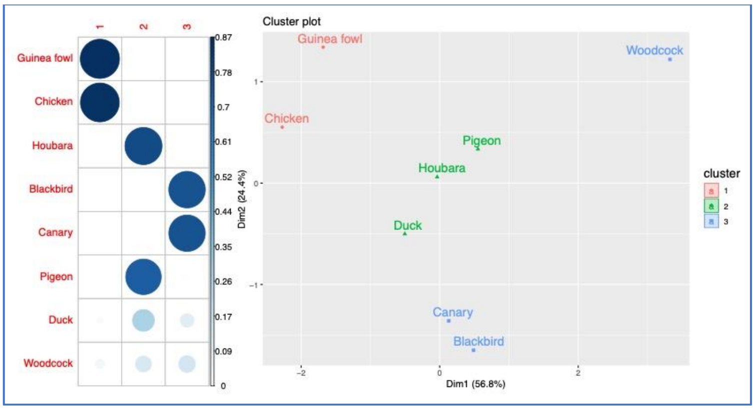


**Supplementary Figure S2.2.** FAC score plot generated for the eight bird species studied using four characteristics: BAC hybridization success rate, and numbers of total, intra- and interchromosomal rearrangements. Left: a matrix sorted in descending order (by the degree of fuzziness of three clusters). Dunn's partition coefficient was used to estimate the fuzziness degree. Right: a PCA ordination diagram resulted from applying the fuzzy clustering method.

*2.3. ALC analyses*

To build a plausible phylogenetic tree for the investigated eight avian species and their five characteristics, we performed the ALC clustering based on Euclidean distance metric and average linkage method (UPGMA). This was followed up by bootstrapping validation generating

Approximately Unbiased (AU) *p*-values and Bootstrap Probability (BP) values and using the pvclust software package for R [73]. If AU values were more than 95%, clusters were deemed significant. Interpretation of optimal cluster number was done using the Elbow method [74]. The produced phylogenetic three (Supplementary Figure S2.3) was concordant with the observed clustering patterns using the other mathematical approaches.

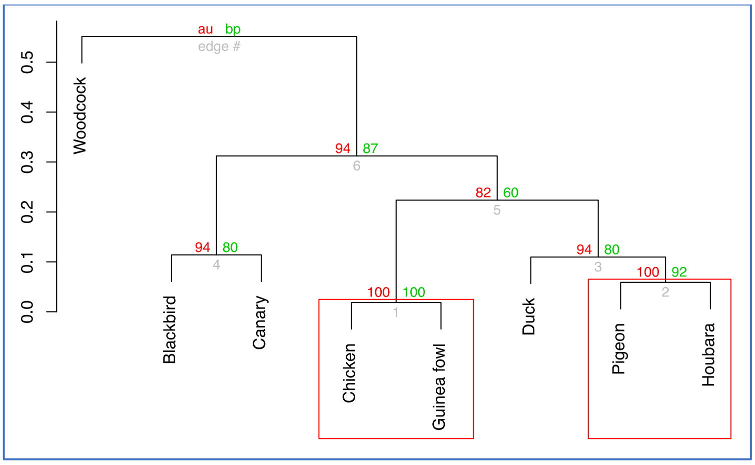


**Supplementary Figure S2.3.** ALC clustering for the eight bird species studied using a matrix of Euclidean distances between objects and based on five characteristics: BAC hybridization success rate, diploid number of chromosomes, and numbers of total, intra- and interchromosomal rearrangements. Bootstrapping validation resulted in AU (Approximately Unbiased) p-values (%) and BP (Bootstrap Probability) values (%) presented as red and green estimates, respectively. Red rectangles contain clusters with AU p-values ≥95%.

Next, we compared the resulting ALC tree (Supplementary Figure S2.3) with the 'reference' one (Figure 1) using the Robinson-Foulds distances between pairs of phylogenetic trees [75–77]. As calculation of the Robinson-Foulds distances showed (Supplementary Figure S2.4), the right ALC-assisted tree did not entirely match the left known phylogeny but was still similar to the clustering patterns obtained with the other approaches.

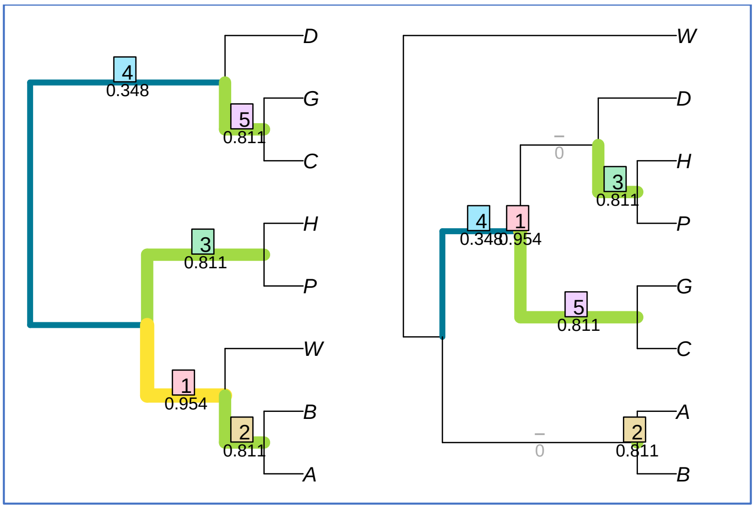

**Supplementary Figure S2.3.** Comparison of the known phylogeny (left) with the one built using ALC and five factors (right), showing the calculated Robinson-Foulds distances between them and suggesting how much there are similarities/differences between the two.

**References**


69 RDocumentation. The R Base Package by R-core R-core@R-project.org; base v3.6.2, 2019; scale function. Available online: https://www.rdocumentation.org/packages/base/versions/3.6.2/topics/scale (accessed 22 December 2020).

70 R: Fuzzy Analysis Clustering; fanny {cluster}. Documentation for package 'cluster' version 2.1.0. "Finding Groups in Data": Cluster Analysis Extended Rousseeuw et al. Available online: https://stat.ethz.ch/R-manual/R-devel/library/cluster/html/fanny.html (accessed 22 December 2020).

71 Shitikov V.K., Mastitsky S.E. (2017) [10.4.2 Fuzzy *k*-means method (fuzzy analysis clustering)], in: [Classification, regression, Data Mining algorithms using R]. Electronic book. Available online: https://ranalytics.github.io/data-mining/104-Other-Clustering-Methods.html#sec_10_4_2 (accessed 22 December 2020).

72 fanny: Fuzzy Analysis Clustering. Documentation for package 'cluster' version 2.1.0, 2019. "Finding Groups in Data": Cluster Analysis Extended Rousseeuw et al. Available online: https://rdrr.io/cran/cluster/man/fanny.html (accessed 22 December 2020).

73 Suzuki, R.; Shimodaira, H. Pvclust: an R package for assessing the uncertainty in hierarchical clustering. *Bioinformatics* **2006**, *22*, 1540–1542. https://doi.org/10.1093/bioinformatics/btl117.

74 Zhao, Q.; Hautamaki, V.; Fränti, P. Knee Point Detection in BIC for Detecting the Number of Clusters. In International Conference on Advanced Concepts for Intelligent Vision Systems – ACIVS 2008, Lecture Notes in Computer Science; Blanc-Talon, J., Bourennane, S., Philips, W., Popescu, D., Scheunders, P., Eds.; Springer: Berlin, Heidelberg, Germany, 2008; Vol. 5259, pp. 664–673, ISBN 978-3-540-88457-6.

75 Smith, M.R. TreeDist: Distances Between Phylogenetic Trees (Version v0.0.0.9000). Zenodo. 2019. https://doi.org/10.5281/zenodo.3528124. Available online: https://zenodo.org/record/3528124 (accessed 24 December 2020).

76 Smith, M.R. Information theoretic Generalized Robinson-Foulds metrics for comparing phylogenetic trees. *Bioinformatics* 2020a, Jul 3:btaa614. https://doi.org/10.1093/bioinformatics/btaa614.

77 Smith, M.R. Visualise a matching – VisualizeMatching. TreeDist 1.2.1. 2020b. Available online: https://ms609.github.io/TreeDist/reference/VisualizeMatching.html (accessed 22 December 2020).
